# Left atrial-left ventricular angle, a new measure of left atrial and left ventricular remodeling

**DOI:** 10.1007/s10554-021-02411-z

**Published:** 2021-09-22

**Authors:** Maha A. Al-Mohaissen, Benjamin J. W. Chow, Terry Lee, Kwan-Leung Chan

**Affiliations:** 1 Department of Clinical Sciences (Cardiology), College of Medicine, Princess Nourah Bint Abdulrahman University, PO Box 84428, Riyadh, 11671 Saudi Arabia; 2 University of Ottawa Heart Institute, Ottawa, ON Canada; 3 Department of Medicine (Cardiology), University of Ottawa, Ottawa, ON Canada; 4 Department of Radiology, University of Ottawa, Ottawa, ON Canada; 5 Centre for Health Evaluation and Outcome Sciences, St. Paul’s Hospital and University of British Columbia, Vancouver, BC Canada

**Keywords:** Left atrium, Left-atrial volume, Left-atrial remodeling, Left atrial–left ventricular angle, Area-length method, Left atrial geometry

## Abstract

We assessed the left atrial-left ventricular (LA-LV) long axis angulation value as a new measure of LA remodeling, and studied its predictors, its effect on two-dimensional LA volume (2D LAVol) estimation, and optimization techniques for 2D LAVol values. Retrospective electrocardiogram-gated coronary computed tomographic angiograms of 164 consecutive patients were reviewed. The LA–LV angle was measured in reconstructed 3-chamber views, and its predictors were determined. The LAVol measured by the area-length method after image optimization along the LV long axis (AL) and the LA long axis (AC–AL), was compared with that measured by the three-dimensional (3D)-volumetric method. LAVol calculation was modified to minimize differences from the 3D values. LA–LV angles ranged from 0° to 63°. In the univariate analysis, decreasing angulation was significantly associated with increasing LV end-diastolic volume (LVEDV), mitral regurgitation grade, LV and LA anteroposterior dimensions, and decreasing LV ejection fraction (LVEF). On multivariate analysis, increasing LVEDV, MR, and LA anteroposterior dimension inversely correlated with angulation; LVEF was positively correlated. The AL and 3D methods significantly differed only for patients with angles ≤ 29.9°. Conversely, LAVol was overestimated for all angules by AC–AL. Modification of AL LAVol using a regression equation, or by substituting the shortest with the longest and average LA lengths in patients with angles ≤ 29.9° and 30–39.9°, respectively neutralized the difference. The LA–LV angle is a new measure of LA and LV remodeling predicted by LV size and function, MR, and LA-anteroposterior dimension. AL formula modifications based on angulation in LV-optimized views better correlate with the 3D method than LA-view modification.

## Introduction

A recent interest in the malalignment of the left atrial (LA) long axis relative to that of the left ventricle (LV) has surfaced, particularly in relation to its potential implications on LA volume (LAVol) determination by two dimensional-transthoracic echocardiography (2D-TTE) [1–3] which has been used to obtain most normative and predictive LAVol data [1, 4, 5]. In the clinical practice, the 4-chamber and 2-chamber 2D-TTE views are routinely optimized along the LV long axis such that an unrecognized angulation during scanning may lead to LA foreshortening and underestimation of LAVol by 2D-TTE [1, 3, 5, 6]. While obtaining extra views along the LA long axis has been advocated [1, 3], the value of this approach and its techniques are yet to be clearly established [1, 7].

Additionally, current knowledge of this parameter is limited and its predictors have not been investigated. It has been speculated that elevation of the LV apex by the diaphragm in older individuals may contribute to increased angulation [8] but no evidence exists on this potential mechanism. Further investigation of this parameter is required as it may add to and improve the present measures of LA remodeling [9, 10] namely 2D LAVol. LA structural remodeling is complex, involves more than enlargement of the LA [11], and is related to LV remodeling [12]. Improved understanding of LA remodeling and its mechanisms provides valuable diagnostic, prognostic, and therapeutic insights into the management of patients with cardiac conditions [13].

Computed tomographic angiography (CTA) enables accurate and validated determination of LAVol [14] and can also determine LA–LV angulation and perform image optimization along the LA long axis with the use of proprietary software. This study aimed to assess the clinical and anatomical predictors of LA–LV angulation, and its effect on LAVol measurement by the standard area-length (AL) method, and techniques for the optimization of AL LAVol calculation based on angulation degree.

## Methods

### Patient population

This study enrolled 164 consecutive patients who had undergone both a clinically indicated retrospective electrocardiogram (ECG)-gated coronary CTA and 2D-TTE within a 15-day period from January 2008 to October 2010 at the Ottawa Heart Institute. These patients were enrolled in a registry and their clinical and imaging data including age, sex, height, weight, LV end-diastolic volume (LVEDV), and LV ejection fraction (LVEF) by CTA, were prospectively recorded. Patients with atrial fibrillation, congenital heart disease, mechanical mitral prosthesis, or history of cardiac transplantation were excluded from the analysis. The baseline characteristics of the participants have been described in a previous study [15]. CTA data of 13 originally registered patients were missing and therefore were excluded from this analysis. The study was approved by the Institutional Human Research Ethics Board.

### Coronary computed tomography angiography data

Coronary CTAs were performed using a GE Volume CT (GE Healthcare, Milwaukee, WI). Retrospective ECG-gated datasets were obtained using 64 × 0.625 mm slice collimation. A single-segment reconstruction algorithm was performed and ten phases (5–95%) with 1.25 mm slice thickness with 0.625 mm increments were reconstructed for the LA measurements.

**Fig. 1:**
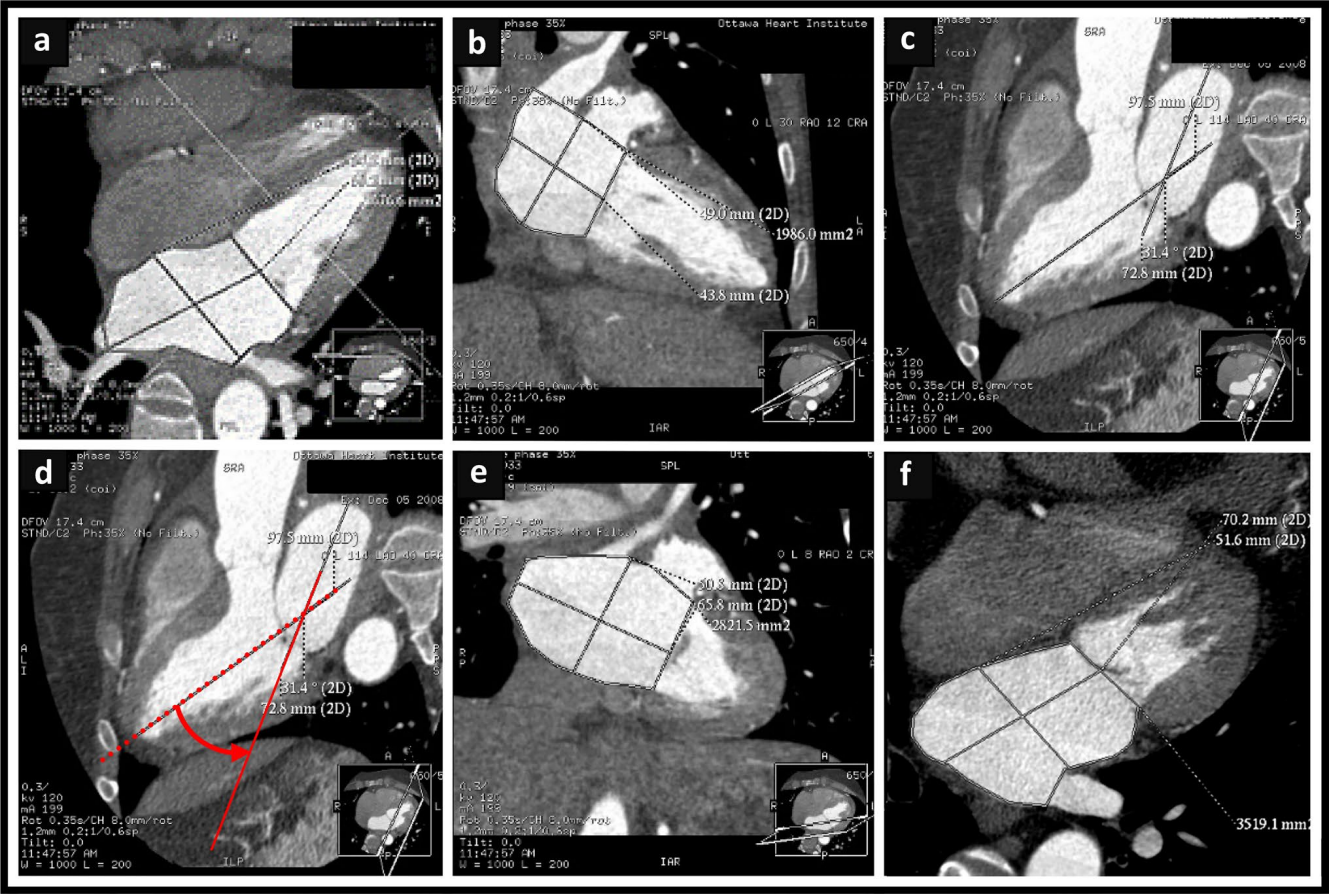
Measurement of LA–LV angulation and LAVol using the AL method before and after correction for LA–LV angulation. Measurement of LAVol using the AL method from images obtained along the long axis of the LV (**a** and **b**). **c** Measurement of LA–LV angle in a reconstructed 3-chamber view using the proprietary software. **d** Image reconstruction for optimization of the LA long axis (solid red line). Acquisition of the new LA dimensions from 2-chamber (**e**) and 4-chamber (**f**) views formatted along the LA long axis according to the LA–LV angle for calculation of the LAVol by the AC–AL. *AC-AL* Angle corrected area-length, *AL* Area length, *LA* Left atrium, *LA-LV* left atrial-left ventricular, *LAVol* Left atrial volume, *LV* Left ventricle

Using CTA, the angulation degree of the LA long axis relative to that of the LV in a reconstructed 3-chamber view was measured using the proprietary software. The LA–LV angle was the intercept angle between the LA long-axis and the LV long-axis, using a view that optimized the LV and LA lengths and demonstrated the LV inflow and outflow tracts akin to the echocardiographic parasternal long-axis view (Fig. 1c).

For each patient, a single reader, blinded to the clinical data, measured LA length, and area in the reconstructed 2- and 4-chamber views using images formatted in the following manner: (1) along the long axis of the LV and (2) along the LA long axis after determination of the LA–LV angle (Fig. 1a–f) with the proprietary software. Additionally, LAVol measured by the CT-three-dimensional (3D) volumetric method was used as the reference standard. A semi-automated software with an attenuation–based endocardial border detection, allowing for manual correction, was used for this purpose with exclusion of the LA appendage and pulmonary veins from the 3D-LAVol measurement as described previously [15]. After acquiring all the data, 2D LAVol was calculated for each participant from the AL formula [5]. Data in the LV optimized views were used to calculate the AL LAVol, while data obtained from the LA optimized views were used to calculate the angle corrected-AL (AC–AL) LAVol.

### 2D-transthoracic echocardiography data

The 2D-TTE of the patients were reviewed. The following were measured once by a single reader blinded to the CTA angle results and in accordance with the current recommendations [5]: the aortic root, the ascending aorta, the LA anteroposterior dimension (LAd), and LV end diastolic dimension (LVEDd) from the parasternal long axis view and right atrial minor axis diameter (RAd) from the apical 4-chamber view. The presence and severity of mitral regurgitation was recorded.

### Statistical analysis

Continuous variables are presented as means with standard deviations and median with interquartile range, and categorical variables are presented as frequencies with percentages. Statistical significance was defined as *P* < 0.05. Demographic variables and LAVols were stratified by LA–LV angle and compared across strata using the Cochran–Armitage or Jonckheere–Terpstra tests as appropriate. The association between LA–LV angles and clinical and anatomical predictors was analyzed using linear regression. Predictors which were significant in the univariate analysis were further included in multivariate linear regression analysis. Difference between the LAVol using 2D and 3D measurements was assessed using a t-test. The Pearson correlation coefficient was also used to assess the association between the 2D and 3D measures. Analyses were performed using SAS 9.4 (SAS Institute Inc., Cary, NC, USA).

## Result

### Values and predictors of LA-LV angles

A total of 164 patients were included in the study. Baseline characteristics of the study population stratified according to the LA–LV angles are summarized in Table 1. Mean age was 58.5 (± 13.8) years and 62.8% were men. The LA–LV angles ranged from 0° to 63° (mean 31.9 ± 12.3°) in this population. A significant decreasing trend was observed for LA–LV angulation and increasing CTA LVEDV (*P* = 0.039), and LA anteroposterior diameter in the 2D-TTE parasternal long axis view (*P* < 0.001) (Table 1). The trend between decreasing LA–LV angulation and 3D LAVol, age, sex, CTA LVEF, body surface area (BSA), body mass index (BMI), and mitral regurgitation grade was nonsignificant. Similarly, the association between the LA–LV angle and other anatomic measures, namely aortic root, ascending aorta, and right atrial and left ventricular end-diastolic dimensions was nonsignificant.

**Table 1:** Baseline characteristics of the study population by left atrial-left ventricular angulation

Variable	All (*n* = 164)	^a^LA-LV angles (°)					
		0–19.9 (*n* = 22)	20–29.9 (*n* = 34)	30–39.9 (*n* = 68)	40–49.9 (*n* = 32)	50 + (*n* = 8)	*P*
LA–LV angles (°)							–
Mean (SD)	32.0 (12.3)	9.1 (6.3)	24.7 (3.1)	34.7 (2.7)	44.0 (2.5)	54.8 (4.3)	
Median (IQR)	33.8 (26.2, 39.3)	8.9 (3.0, 14.0)	25.1 (21.2, 27.0)	35.0 (32.1, 36.8)	44.3 (42.0, 45.8)	54.0 (51.4, 57.3)	
Range	(0.0, 63.0)	(0.0, 19.7)	(20.0, 29.9)	(30.0, 39.4)	(40.0, 48.6)	(50.0, 63.0)	
Clinical characteristics							
Age, years							0.730
Mean (SD)	58.5 (13.8)	59.9 (11.8)	59.9 (15.8)	56.9 (14.1)	58.4 (13.2)	62.6 (11.7)	
Range	(19.0, 88.0)	(35.0, 84.0)	(19.0, 84.0)	(27.0, 88.0)	(34.0, 79.0)	(38.0, 76.0)	
Male sex, *n* (%)	103 (62.8)	13 (59.1)	19 (55.9)	46 (67.6)	23 (71.9)	2 (25.0)	0.938
BMI (kg/m^2^)^b^							0.234
Mean (SD)	28.9 (5.9)	31.2 (8.4)	28.4 (4.9)	29.1 (5.4)	27.8 (5.9)	28.6 (5.5)	
Range	(17.4, 49.7)	(19.8, 49.7)	(21.6, 42.1)	(17.4, 43.3)	(20.4, 45.1)	(24.3, 40.6)	
BSA (m^2^)^c^							0.855
Mean (SD)	1.95 (0.27)	2.02 (0.37)	1.90 (0.24)	1.96 (0.26)	1.94 (0.25)	1.91 (0.21)	
Range	(1.40, 2.95)	(1.48, 2.95)	(1.45, 2.40)	(1.40, 2.71)	(1.47, 2.73)	(1.66, 2.15)	
Indications for CTA^d^							
Chest pain, *n* (%)	91 (55.5)	10 (45.5)	19 (55.9)	41 (60.3)	15 (46.9)	6 (75.0)	0.498
Palpitations, *n* (%)	90 (54.9)	15 (68.2)	16 (47.1)	39 (57.4)	14 (43.8)	6 (75.0)	0.604
Heart failure, *n* (%)	18 (11.0)	5 (22.7)	4 (11.8)	5 (7.4)	3 (9.4)	1 (12.5)	0.196
Valvular heart disease, *n* (%)	30 (18.3)	3 (13.6)	6 (17.6)	11 (16.2)	8 (25.0)	2 (25.0)	0.335
CTA variables							
LVEF (%)^e^							0.056
Mean (SD)	60.5 (13.5)	51.1 (16.0)	61.7 (15.4)	62.0 (11.7)	60.9 (11.3)	68.3 (10.1)	
Range	(13, 90)	(23, 76)	(21, 80)	(30, 90)	(13, 76)	(50, 81)	
LVEDV (ml)^f^							0.039
Mean (SD)	151.8 (54.3)	202.0 (78.8)	144.3 (46.0)	144.7 (44.4)	144.5 (47.5)	132.8 (41.5)	
Range	(57.2, 357)	(93, 329)	(88, 274)	(57.2, 303)	(93, 357)	(76, 187)	
3D^g^ LAVol (ml)^h^							0.170
Mean (SD)	106.9 (36.7)	122.3 (38.6)	100.4 (33.5)	108.6 (39.8)	100.8 (31.4)	102.9 (30.0)	
Range	(46.4, 297.8)	(74.6, 226.4)	(52.4, 165.0)	(52.7, 297.8)	(47.7, 197.0)	(46.4, 149.0)	
Indexed 3D LAVol (ml/m^2^)							0.184
Mean (SD)	54.9 (16.8)	61.2 (18.0)	52.8 (15.3)	55.3 (17.7)	52.1 (15.2)	54.1 (16.2)	
Range	(27.1, 132.4)	(35.5, 105.8)	(29.5, 80.5)	(27.6, 132.4)	(27.7, 107.7)	(27.1, 73.5)	
2D-TTE variables^i^							
LAd (cm)^j^							<0.001
Mean (SD)	3.94 (0.65)	4.52 (0.62)	3.97 (0.57)	3.83 (0.66)	3.79 (0.55)	3.79 (0.48)	
Range	(2.50, 5.80)	(3.40, 5.80)	(2.80, 5.10)	(2.50, 5.40)	(2.80, 4.90)	(3.10, 4.40)	
LVEDd (cm)^k^							0.093
Mean (SD)	4.95 (0.71)	5.39 (0.97)	4.90 (0.66)	4.88 (0.63)	4.88 (0.72)	4.80 (0.37)	
Range	(3.60, 7.50)	(4.10, 7.20)	(3.60, 6.20)	(3.70, 7.50)	(3.80, 7.10)	(4.30, 5.20)	
ARd (cm)^l^							0.303
Mean (SD)	3.31 (0.42)	3.29 (0.44)	3.24 (0.32)	3.33 (0.44)	3.41 (0.42)	3.16 (0.54)	
Range	(2.40, 4.30)	(2.70, 4.20)	(2.70, 4.00)	(2.40, 4.30)	(2.60, 4.20)	(2.50, 4.10)	
AAd^m^^(cm)							0.289
Mean (SD)	3.24 (0.48)	3.09 (0.31)	3.26 (0.44)	3.23 (0.48)	3.32 (0.61)	3.30 (0.39)	
Range	(2.40, 5.50)	(2.70, 3.70)	(2.40, 4.30)	(2.40, 5.00)	(2.40, 5.50)	(3.00, 4.10)	
RAd (cm)^n^							0.309
Mean (SD)	3.90 (0.77)	4.18 (0.74)	3.87 (0.89)	3.88 (0.67)	3.76 (0.84)	4.05 (0.79)	
Range	(1.50, 6.50)	(2.90, 5.40)	(2.30, 6.50)	(2.60, 6.00)	(1.50, 5.70)	(3.20, 5.10)	
MR grade, *n* (%)^o^							0.081
Absent or mild (≤1)	147 (90.2)	15 (68.2)	33 (97.1)	63 (94)	29 (90.6)	7 (87.5)	
Moderate or moderate-severe (2 and 3)	16 (9.8)	7 (31.8)	1 (2.9)	4 (6.0)	3 (9.4)	1 (12.5)	

*P* value is based on Cochran–Armitage trend test for categorical variable and Jonckheere–Terpstra test for continuous variable and is for testing if there was an increasing (or decreasing) trend with angulation

^a^left atrial-left ventricular, ^b^body mass index, ^c^body surface area, ^d^computed tomographic angiography, ^e^left ventricular ejection fraction, ^f^left ventricular end diastolic volume, ^g^3-dimensional, ^h^left atrial volume, ^i^two-dimensional-transthoracic echocardiography, ^j^anteroposterior left atrial dimension, ^k^anteroposterior left ventricular end diastolic dimension, ^l^aortic root diameter, ^m^ascending aorta diameter, ^^^measured in 160 participants, ^n^right atrial minor axis dimension, ^o^mitral regurgitation

Tables 2 and 3 show the association between LA–LV angles and the studied variables using univariate and multivariate analyses, respectively. A significant inverse correlation was observed from the univariate analysis between LVEDV, mitral regurgitation grade and anteroposterior LAd and LVEDd, with LA–LV angles. Conversely, a positive correlation between LVEF and LA–LV angulation was detected (Table 2). On multivariate analysis, only increasing LVEDV, LVEF, mitral regurgitation grade and LAd independently correlated with LA–LV angulation (Table 3). Each of the statistically significant LV parameters (LVEF, LVEDV and LVEDd) was evaluated in a separate multivariate analysis model. Although these parameters measure different aspect of the LV, they were highly colinear; therefore only one was evaluated at a time.

**Table 2:** Association between LA–LV angulation and the studied variables using linear regression analysis

Variable	Univariate analysis	
	Difference in angulation (95% CI)^m^	*P*
Age (per 5 year increase)	−0.36 (−1.05, 0.32)	0.299
BMI^a^ (per 5 kg/m^2^ increase)	−1.45 (−3.05, 0.15)	0.076
Male	0.69 (−3.24, 4.62)	0.731
BSA^b^ (per 0.1 m^2^ increase)	−0.16 (−0.86, 0.54)	0.652
LVEF^c^ (per 5% increase)	1.27 (0.59, 1.96)	<0.001
LVEDV^d^ (per 5 ml increase)	−0.33 (−0.50, −0.16)	<0.001
Ind. 3D^e^LAVol^f^ (per 5 ml/m^2^ increase)	−0.44 (−1.00, 0.13)	0.127
ARd^g^ (per 1 mm increase)	0.24 (−0.21, 0.69)	0.290
AAd^h^ (per 1 mm increase)	0.31 (−0.09, 0.71)	0.122
RAd^i^ (per 1 mm increase)	−0.18 (−0.43, 0.06)	0.142
LVEDd^j^ (per 1 mm increase)	−0.35 (−0.61, −0.09)	0.009
LAd^k^ (per 1 mm increase)	−0.59 (−0.87, −0.31)	<0.001
MR^l^ grade (≥ moderate vs absent or mild)	−9.09 (−15.36, −2.82)	0.005

Values presented are estimated difference in angulation when the particular variable increases or compared to the other level of that variable

^a^body mass index, ^b^body surface area, ^c^left ventricular ejection fraction, ^d^left ventricular end diastolic volume, ^e^3-dimensional, ^f^left atrial volume, ^g^aortic root diameter, ^h^ascending aorta diameter, ^i^right atrial minor axis dimension, ^j^left ventricular end diastolic dimension, ^k^left atrial anteroposterior dimension, ^l^mitral regurgitation, ^m^confidence interval

**Table 3:** Association between LA–LV angles and variables which were significant univariately using multivariate linear regression analysis

Variable	Model 1		Model 2		Model3	
	Difference in angulation (95% CI)^f^	*P*	Difference in angulation (95% CI)	*P*	Difference in angulation (95% CI)	*P*
LVEF^a^ (per 5% increase)	0.89 (0.20, 1.59)	0.012	–		–	
LVEDV^b^ (per 5 ml increase)	–		−0.21 (−0.40, −0.03)	0.026	–	
LVEDd^c^ (per 1 mm increase)	–		–		−0.16 (−0.43, 0.11)	0.240
LAd^d^ (per 1 mm increase)	−0.45 (−0.74, −0.16)	0.003	−0.38 (−0.70, −0.07)	0.018	−0.48 (−0.78, −0.18)	0.002
MR^e^ (≥ moderate vs. absent/mild)	−7.03 (−13.05, −1.02)	0.022	−7.76 (−13.78, −1.74)	0.012	−7.55 (−13.60, −1.49)	0.015

Values presented are estimated difference in angulation when the particular variable increases or compared to the other level of that variable

The multivariate models have R^2^ ranged from 0.14 to 0.17 and all have *p* < 0.001 for the omnibus F test

^a^left ventricular ejection fraction, ^b^left ventricular end diastolic volume, ^c^left ventricular end diastolic dimension, ^d^left atrial anteroposterior dimension, ^e^mitral regurgitation, ^f^confidence interval

### Effect of LA-LV angulation on LAVol measurement by the AL method

Table 4 shows the difference in LAVol between the studied 2D measures and 3D volumetric method, categorized by the degrees of angulation. Results of the attempts on minimizing this difference by adjusting the formula are also shown. A significant difference between the standard AL[5] and 3D was detected (2.6 ml [SD: 1.5, 3.8], *P* < 0.001) in the overall population, but in the angulation subgroups, the difference was only significant in individuals with LA–LV angles between 0° and 29.9° (*P* < 0.001). In the hybrid AL, recalculating LAVol by substituting the longest [16] and average [17] for the shortest LA length in the formula for those with angles ≤ 29.9° and 30–39.9° respectively resolved the difference (*P* = 0.698, Table 4). Similarly, modification of the AL LAVol values according to the individual’s angulation degree by applying an equation obtained by linear regression (Reg) using 3D LAVol as the dependent variable (AL (Reg) = 5.0341 + 0.1255 LA–LV angle + 0.7848 AL LAVol) neutralized the difference in all participants (*P* = 0.063).

**Table 4:** Differences in LAVol measurements between the studied 2-dimensional and 3-dimensional methods

Comparison	All (*n* = 164)	^a^LA–LV angles (°)				
		0–19.9 (*n* = 22)	20–29.9 (*n* = 34)	30–39.9 (*n* = 68)	40–49.9 (*n* = 32)	50 + (*n* = 8)
*StandardAL*^*b*^ *and AL modifications vs 3D*^*c*^						
Standard AL (Min. LA length)^d^ vs. 3D (ml^3^)						
Mean difference (95% CI)	2.6 (1.5, 3.8)	5.8 (3.0, 8.6)	5.2 (3.1, 7.4)	1.5 (−0.4, 3.4)	1.3 (−1.3, 3.8)	−2.0 (−9.9, 5.8)
Range	(−15.2, 19.7)	(−7.2, 19.7)	(−7.5, 18.7)	(−15.0, 18.2)	(−15.2, 15.6)	(−14.8, 14.1)
* P*	<0.001	<0.001	<0.001	0.115	0.319	0.562
AL (Max. LA length)^e^ vs. 3D (ml^3^)						
Mean difference (95% CI)	−2.4 (−3.7, −1.1)	1.8 (−0.7, 4.2)	1.8 (−0.5, 4.2)	−3.6 (−5.6, −1.6)	−5.2 (−8.4, −2.0)	−9.9 (−21.0, 1.2)
Range	(−30.8, 13.5)	(−11.9, 13.5)	(−12.2, 12.6)	(−21.2, 10.2)	(−30.8, 12.2)	(−30.3, 3.9)
* P*	<0.001	0.152	0.125	0.001	0.002	0.074
AL (Av. LA length)^f^ vs. 3D (ml^3^)						
Mean difference (95% CI)	−0.1 (−1.3, 1.1)	3.7 (1.2, 6.2)	3.4 (1.2, 5.7)	−1.3 (−3.2, 0.6)	−2.4 (−5.3, 0.5)	−6.5 (−16.2, 3.3)
Range	(−25.4, 16.5)	(−9.7, 16.5)	(−9.0, 15.0)	(−16.3, 11.8)	(−25.4, 13.9)	(−23.8, 8.3)
* P*	0.845	0.006	0.003	0.168	0.097	0.159
AL (Hybrid) vs. 3D (ml^3^)						
Mean difference (95% CI)	0.2 (−0.9, 1.3)	1.8 (−0.7, 4.2)	1.8 (−0.5, 4.2)	−1.3 (−3.2, 0.6)	1.3 (−1.3, 3.8)	−2.0 (−9.9, 5.8)
Range	(−16.3, 15.6)	(−11.9, 13.5)	(−12.2, 12.6)	(−16.3, 11.8)	(−15.2, 15.6)	(−14.8, 14.1)
* P*	0.698	0.152	0.125	0.168	0.319	0.562
AL (Reg) ^ vs. 3D						
N	82	11	15	42	11	3
Mean difference (95% CI)	−1.4 (−2.8, 0.1)	−4.2 (−8.7, 0.4)	0.4 (−3.5, 4.4)	−1.6 (−3.8, 0.6)	−1.5 (−3.5, 0.5)	3.2 (−9.0, 15.4)
Range	(−15.7, 15.1)	(−15.7, 8.1)	(−11.2, 15.1)	(−15.0, 12.5)	(−6.7, 1.9)	(−0.5, 8.8)
* P*	0.063	0.068	0.814	0.152	0.131	0.381
*AC-AL*^*g*^ *and modifications vs 3D*						
AC-AL vs. 3D (ml^3^)						
Mean difference (95% CI)	13.0 (11.8, 14.1)	7.0 (4.2, 9.7)	11.6 (9.3, 14.0)	14.0 (12.3, 15.6)	16.1 (13.0, 19.3)	14.0 (7.3, 20.8)
Range	(−7.2, 46.8)	(−7.2, 23.8)	(−0.8, 27.2)	(−5.0, 31.1)	(2.5, 46.8)	(2.6, 28.2)
* P*	<0.001	<0.001	<0.001	<0.001	<0.001	0.002
AC-AL (Max. LA length) vs. 3D (ml^3^)						
Mean difference (95% CI)	8.1 (7.1, 9.1)	4.2 (1.7, 6.7)	7.2 (5.0, 9.4)	8.5 (7.0, 9.9)	10.9 (8.6, 13.2)	9.1 (4.2, 13.9)
Range	(−11.9, 29.1)	(−11.9, 13.1)	(−3.2, 25.6)	(−6.7, 19.9)	(−1.7, 29.1)	(0.4, 18.8)
* P*	<0.001	0.002	<0.001	<0.001	<0.001	0.003

^Data was randomly split into two equal parts as training set and validation set. Angle corrected volume was obtained by the Eq. 5.0341 + 0.1255 LA-LV angle + 0.7848 AL LA vol. The equation was obtained by linear regression using 3D LAVol as the dependent variable from the training data set. Difference between the estimate volume and 3D was based on the validation set

All LAVol are indexed to body surface area

*P* value is based on t-test and is for comparing the difference between volume measurements within each angulation group

^a^left atrial-left ventricular, ^b^area-length, ^c^3-dimensional, ^d^minimum, ^e^maximum, ^f^average, ^g^angle corrected area-length

**Fig. 2:**
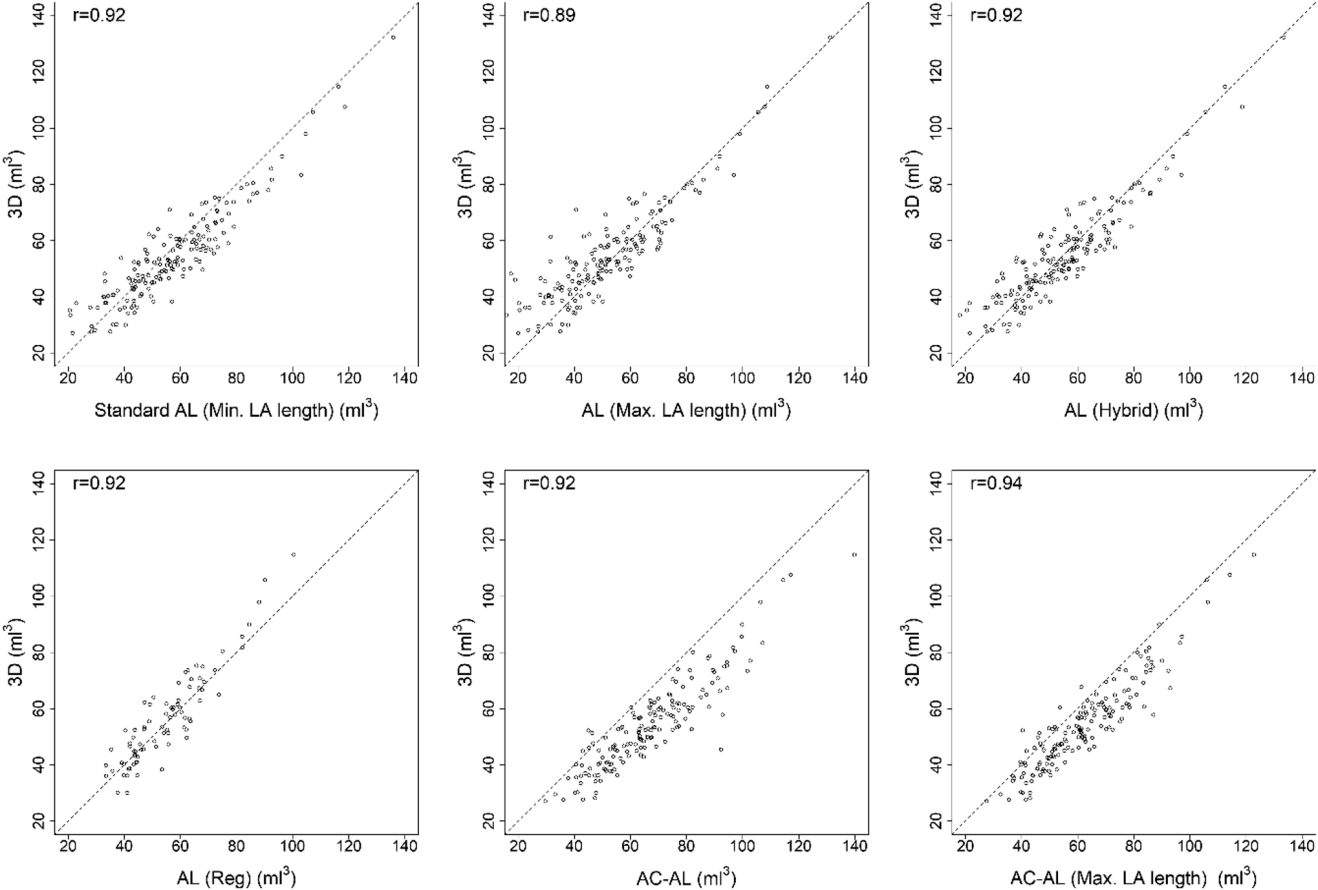
Scatterplot and correlation between LAVol by the studied 2D and 3D methods using the Pearson correlation. The dashed line is the diagonal line representing the equivalence of the 2D measurements and the 3D. All LAVols are indexed to the body surface area. *AC*–*AL* Angle corrected area-length, *AL* Area-length, *3D* 3-dimensional, *LA* Left atrium, *Max*. Maximum, *Min.* Minimum, *r* Pearson correlation, *Reg* Regression

Conversely, LAVol was significantly overestimated by AC-AL compared with 3D at all degrees of LA–LV angulation (*P* < 0.001). Reassessment by dividing by the longest LA length (AC-AL, Max LA length) in all participants decreased the difference, although it was significant (*P* < 0.001) (Table 4). Overall, correlation (r ≥ 0.89) of all the studied 2D LAVol measures with the reference standard (Fig. 2) was strong.

## Discussion

This study shows that the angle at which the LA intersects the LV varies among individuals, and in this patient population, it ranged from 0° to 63° (mean 31.9 ± 12.3°). Among the studied determinants of LA–LV angulation, significant trends for increasing LVEDV, and LAd were observed with decreasing angulation. Additionally, based on the univariate analysis, a significant positive correlation with LVEF and a significant inverse correlations with LVEDd, LAd, and mitral regurgitation grade were observed. On multivariate analysis, LVEF, LVEDV, LAd and mitral regurgitation grade were independently associated with LA–LV angulation. No significant association was noted for the participants’ age, sex, or diameters of the aortic root, ascending aorta, and right atrium.

We found that only in subjects with small LA–LV angles (0–29.9°), a significant difference existed between standard AL and 3D LAVols with no significant difference in those with angulations ≥ 30.0°. Conversely, LAVol determined by the AC–AL method (image optimization along the LA long axis) was significantly greater than that by the 3D method at all degrees of angulation. Modification of the AL formula, by substituting the shortest by the longest [16] and average [17] LA lengths for those with angulations ≤ 29.9°, and 30–39.9°, respectively (hybrid AL) and modification of the AL LAVols with a regression equation obliterated the difference in all participants. Overall, a strong correlation for all the studied 2D LAVol measurement methods was noted with the reference standard. To the best of our knowledge, the predictors of LA-LV angulation and its effect on LAVol measurement have not been studied previously, and this is the first study to measure and report on this parameter.

### LA–LV angulation and its determinants

Decreased LA-LV angulation may be a new additional measure of LA remodeling [9, 18, 19], beyond LAVol, in patients with mitral regurgitation and LV enlargement and dysfunction, and relate LA to LV remodeling [12]. Unlike LAVol, LA–LV angulation was not associated with the participants’ age, sex, or BSA, although both measures related to LVEDV [20], contrary to LA sphericity, LA–LV angulation was not associated with 3D LA volume or the patients sex [9].

A reduced LVEF, increased LVEDV, anteroposterior LA dimension and mitral regurgitation grade were independent predictors of LA–LV angulation in this study. This constellation of cardiac pathology is commonly observed in patients with heart failure and reduced ejection fraction who have enlarged dysfunctional ventricles [21], greater mitral regurgitation [22] and eccentric LA remodeling [23]. A greater association between LA–LV angulation and LAd compared with 3D LAVol was observed. LA enlargement is not symmetrical [24] and the effect of increasing LA size on decreasing angulation appears primarily driven by increases in LAd. The changes in LA–LV angulation may additionally relate to changes in the mitral annular plane. A recent study involving patients with atrial dilatation and atrial functional mitral regurgitation identified horizontal inclination of the mitral annular plane that decreased with surgical plication, along with decrease of the LA dimension and improvement of the regurgitation [25]. The significant association between LA–LV angulation and worsening mitral regurgitation supports this possibility.

The mechanisms behind the changes in angulation in particular are not apparent. There is paucity of data on the geometric interaction of the cardiac chambers in disease states. Anatomically, the LA forms most of the heart’s base and joins the base of the LV at the mitral valve orifice, anteroinferiorly and to the left [26]. The left ventricle in turn slopes from its base in the plane of the atrioventricular groove to the cardiac apex [27]. It may be postulated that some degree of angulation normally exists in the longitudinal axis of the two chambers and with increasing LV volume and displacement, as well as LA remodeling the angulation decreases.

Although increases in LA–LV angulation have been speculated to occur with age [8], we did not observe such an association in this study. All these mechanistic hypotheses including the potential role of thoracic constraints remain to be explored [9].

### Effect of LA-LV angulation on LAVol measurement

The standard AL method overestimated mean indexed LAVol by 2.6 ml compared with 3D but the difference was significant only in patients with angles ≤ 20–29.9°. Discrepancies between 2D and 3D LAVols assessed by the same imaging modality have been reported by other investigators, CTA data are, however, limited [15]. Echocardiographic comparisons have shown both significantly larger [3] and smaller [28] 3D LAVols compared to those obtained by 2D. These differences may be attributed to the type of software used, endocardial tracing errors, 2D misalignment of orthogonal apical 4-chamber and apical 2-chamber views [2] and 2D method used, with a tendency of the AL formula to yield larger volumes [29]. Conversely, the AC-AL method significantly overestimated the mean indexed LAVol by 13 ml compared with 3D and at all angles; with the largest difference (16.1 ml) observed in those with angles 40–49.9°. Comparisons of 2D- and 3D-TTE LAVols, obtained with image optimization along LA long axis, have shown smaller 2D LAVol (with the method of discs), compared with 3D. With 2D-TTE however, and despite all attempts, LA foreshortening and underestimation of LA size may be unavoidable due to the constrains related to acoustic access and the lack of a reliable way to verify and exclude LA foreshortening [3]. CTA overcomes most of these limitations which renders it better suited for 2D LAVol validation.

Among the 2D methods, the standard AL method appears to correlate best with 3D [15]. Despite its excellent performance, however, the AL formula has its inherent limitations and may not be universally applicable. In our study, the formula served best at higher angulation degrees but significantly overestimated LAVol in those with LA–LV angles below 30°. Correcting the results of the AL formula by a regression equation or applying the longest, shortest, or average obtained LA length in the AL formula based on angulation degree, resolved this difference, while maintaining the strong correlation with 3D. Both the longest [16] and average [17] atrial lengths have been used previously.The finding that the AL formula performs differently at different angles suggests that the methods used for 2D LAVol assessment may need to be individualized. The Simpson’s and AL methods are highly correlated [17]. Therefore, the validity of the Simpson’s method in atrial optimized views cannot be speculated and verification is warranted as many clinical [30] and prognostic [31–33] decisions rely on the LAVol.

The normal values for LAVol have been increased in the recent guidelines based on new, larger volume, and prognostic data [5]. However, it is not clear if the increased cutoff values are due to larger data, or among other, shifting to atrial focused views. LAVols in studies utilizing standard LV [34, 35] and atrial [36] optimized views, appear comparable, and different values were obtained with non-foreshortened atrial views [28, 37]. The lack of use of a standardized imaging technique in dedicated atrial imaging may have contributed to this discrepancy, particularly as atrial optimization was achieved by maximizing LA area [36], length [28, 37], length and base [3] and obtaining atrial focused views in LV optimized images [38, 39].

A small study (*n* = 30), assessed LAVol by 2D-TTE from both atrial non-foreshortened and standard apical views with comparison with 3D-TTE. Larger LAVols were obtained from the atrial views, and had better correlation with those from 3D-TTE [40]. Similarly, larger LAVols using the angle corrected view and with a high correlation with those from the 3D method were noted in our study, although these values were significantly different from those of the gold standard. In our study, the comparison of LAVol values in quartiles of angulation provided additional clarification. Our comparison, in addition to the larger sample size, has the advantage of the higher spatial resolution of CTA and better control on image formatting as the 2D-TTE machines are not yet equipped with the software we have used. Given the lack of such technology, if LAVol on 2D-TTE is discrepant from the patient’s clinical background, visual estimation of the LA–LV angle in the parasternal long axis, or apical three-chamber view may explain the findings. Modification of the formula instead of the views in 2D-TTE, is expected to avoid the technical complexity, afford greater consistency and reproducibility, as the atrial views have been associated with an interobserver bias [3], and permit retrospective determination of LAVol from standardized 2D-TTE views [34].

In conclusion, LA–LV angulation appears to be a new promising measure of LA and LV geometry that may be useful in the assessment of suspected cardiac disease. LAVol, among others, is affected by the anatomy of the individual, formula used for its calculation, and the operator’s scanning angle, individually or in combination. The effect of LA–LV angulation on the standard AL derived LAVol though statistically significant may not warrant obtaining extra images along the LA long axis. Such approach may be time consuming, and associated with measurement bias and overestimation of LAVol. Instead, adjusting the AL formula or LAVol values based on angulation degree yields more accurate results compared to those from 3D. Further studies in larger populations are required to validate our observations.

### Study limitations

The measurements for LAVol were performed by a single reader. A bias may have occurred due to overestimation of the AC–AL components, and consequently, AC LAVols. This situation however reflects an everyday practice, where different scanners and readers perform the measurements and obtaining views off the standard LV axis maybe difficult and may not be reproducible, particularly for the follow up of patients. Additionally, we measured LA–LV angulation in a single plane, and only have information of the anteroposterior plane whereas the LA has a 3D orientation. Studies assessing the associations and effects of LA–LV angulation on LAVol in larger populations are required to validate our observations.

## Data Availability

Data are available upon reasonable request.
